# Excitatory neuronal connectivity in the barrel cortex

**DOI:** 10.3389/fnana.2012.00024

**Published:** 2012-07-11

**Authors:** Dirk Feldmeyer

**Affiliations:** ^1^Institute of Neuroscience and Medicine, INM-2, Research Centre JülichJülich, Germany; ^2^Department of Psychiatry, Psychotherapy, and Psychosomatics, RWTH Aachen University, and Jülich-Aachen Research Alliance-Brain, Translational Brain MedicineAachen, Germany

**Keywords:** barrel cortex, cortical column, excitatory connections, long-range collaterals, pyramidal cell, somatosensory cortex, spiny stellate cell

## Abstract

Neocortical areas are believed to be organized into vertical modules, the cortical columns, and the horizontal layers 1–6. In the somatosensory barrel cortex these columns are defined by the readily discernible barrel structure in layer 4. Information processing in the neocortex occurs along vertical and horizontal axes, thereby linking individual barrel-related columns via axons running through the different cortical layers of the barrel cortex. Long-range signaling occurs within the neocortical layers but also through axons projecting through the white matter to other neocortical areas and subcortical brain regions. Because of the ease of identification of barrel-related columns, the rodent barrel cortex has become a prototypical system to study the interactions between different neuronal connections within a sensory cortical area and between this area and other cortical as well subcortical regions. Such interactions will be discussed specifically for the feed-forward and feedback loops between the somatosensory and the somatomotor cortices as well as the different thalamic nuclei. In addition, recent advances concerning the morphological characteristics of excitatory neurons and their impact on the synaptic connectivity patterns and signaling properties of neuronal microcircuits in the whisker-related somatosensory cortex will be reviewed. In this context, their relationship between the structural properties of barrel-related columns and their function as a module in vertical synaptic signaling in the whisker-related cortical areas will be discussed.

## Introduction

In the 1950s, Vernon Mountcastle (Mountcastle, 1957, 1997, 2003) introduced the expression “cortical column” for the concept of vertical information processing in the somatosensory cortex, an idea that was later adopted by David Hubel and Torsten Wiesel (Hubel and Wiesel, 1959, 1963) for the visual cortex. However, in recent years the existence of such vertical modules of cortical signal processing has become a matter of scientific debate. Some reviews and/or commentaries have proposed that the “cortical column” is “a structure without function” (Horton and Adams, 2005) and obituaries for cortical columns have also been written (da Costa and Martin, 2010; but see Rockland, 2010).

The ground-breaking work by Woolsey and Van der Loos (Woolsey and Van der Loos, 1970) showed that the vibrissae on the rodents' snout are topographically represented in the contralateral somatosensory cortex by distinct cytoarchitectonic units in layer 4. These cytoarchitectonic units have therefore been coined “barrels” to describe their structure and the cortical region in which they are located as “barrel field.” Already in 1922, Lorente de Nó (Lorente de Nó, 1922; for a translation see Lorente de Nó, 1992) showed such barrel-like structures (see Figures 5–8 in Lorente de Nó, 1922, 1992) which he assumed were located in the acoustic cortex.

Here, aggregations of somata of small spiny neurons exist that surround a “hollow” center. In the mouse and many other rodents “hollows” are clearly visible for every barrel (Woolsey et al., 1975a) while in the rat such hollows are only discernible in the anterolateral, large barrels (Welker and Woolsey, 1974; Land and Erickson, 2005). The cell density in the barrel hollows is lower than in the barrel borders; barrel hollows contain a large fraction of the thalamocortical and intracortical axons, dendrites, the somata of some L4 neurons and possibly also glia (see e.g., Woolsey et al., 1975a; Lübke et al., 2000). Barrels are separated by narrow septa (Woolsey and Van der Loos, 1970; Welker and Woolsey, 1974) which are narrower in mice than in rats (Woolsey et al., 1975b). Distinct cortical microcircuits have been proposed for barrel- and septum-related excitatory neurons (see e.g., Alloway, 2008). However, since the intracortical microcircuits of septum-related spiny neurons are not known they will not be discussed in detail in this review.

Barrel- and septum-related cortical columns (from layer 1–6) are defined by the barrel and septum borders in layer 4, in the framework of which the synaptic connectivity will be discussed here. For the different cortical layers the following definitions will be used: Layer 1 (L1), layer 2/3 (L2/3) with a distinction between layer 2 (L2) and 3 (L3), layer 4 (L4), layer 5 (L5) with its sublaminae 5A (L5A) and 5B (L5A) and layer 6 (L6) with the sublaminae 6A (L6A) and 6B (L6B). This terminology has been introduced by Lorente de Nó (Lorente de Nó, 1922) for the mouse and adopted by Valverde (Valverde et al., 1989) for the rat and has been used in many other publications. I will also use this nomenclature for the discussion of synaptic connectivity patterns in the somatosensory barrel cortex.

It should be noted, however, that the boundaries of the different layers are often not very sharp and dependent on the type of staining and observation method used. Several different characteristics can be used in combination to define the cortical lamination. The density and distribution of excitatory neurons are clearly one of them and have been used already in early publications on the organization of the neocortex (Ramón y Cajal, 1904; Lorente de Nó, 1922). However, the changes in cell density between the cortical layers are generally gradual, particular those between layer 5 and 5B as well as 6A and 6B (for the barrel cortex see Meyer et al., 2010b). Nevertheless, some layers are readily identifiable such a cortical layers 1 and 4 because of their low and high cell density, respectively; this feature is even visible in unstained neocortical slices (e.g., Marx et al., 2012). At some layer borders neuronal cell types change abruptly, for example at the border between layer 4 and 5A: while layer 4 contains only spiny stellate cells and star pyramids, exclusively slender-tufted pyramidal cells are found immediately below the layer border (e.g., Lübke et al., 2000).

Another feature that helps to delineate cortical layers in the barrel cortex is the projection pattern of the afferents from the ventroposterior medial (VPM) and the posteromedial (POm) thalamic nuclei, which have distinct and generally no-overlapping target regions (for a qualitative analysis in the barrel cortex see Meyer et al., 2010a; for a review see Ahissar and Staiger, 2010). In addition, the distribution of cortical interneurons differs also markedly between layer (see below) and can help to define cortical lamination.

According to a very recent study on rat barrel cortex, about 88–89% of the 19,000 neurons in a barrel-related column are excitatory neurons while only 11–12% are GABAergic interneuron; thus there are about 2200 interneurons per barrel column. The relative fraction of interneurons differed between cortical layers and sublaminae but was for all layers significantly lower than previously estimated (Meyer et al., 2010b, 2011). The highest fraction of interneurons was found in layers 2 and 5A. In mouse barrel cortex, the total number of neurons in a barrel-related column is only about a third of the value observed for the rat (~6500 neurons) of which 11% are inhibitory interneuron (Lefort et al., 2009), a fraction similar to that found for the rat.

Somatotopic representations of peripheral sensory receptors analogous to those in the rodent barrel cortex have also been identified for other animals. A very prominent example is the star-nosed mole [(Catania et al., 1993; Catania and Kaas, 1995) see also (Fox, 2008) for a comprehensive overview] in which the arrangement of the somatosensory receptors on the animal's nose are reflected in their neocortical representation. However, rodents are much more readily available as experimental animals. For this reason, the barrel cortex has become a model system to study the structural and functional characteristics of cortical neuronal microcircuits. Because of their almost cylindrical arrangement, layer 4 barrel columns are now considered to be “prototypical” cortical columns. Here, I will discuss how the intracortical, thalamocortical, and corticothalamic connectivity patterns in barrel-related cortical columns govern and modulate neuronal signaling.

## Vertical organization of thalamocortical projections in the barrel cortex

Sensory signals from the whiskers on the rodent's snout reach the somatosensory “barrel” cortex *via* several distinct pathways (Table 1). Neurons of the trigeminal ganglion innervate whisker follicles in the skin of the rodent's snout and project to four different trigeminal nuclei in the brainstem. In the brainstem trigeminal complex, rod-shaped cytoarchitectonic units termed “barrelettes” have been identified that show a somatotopic arrangement reflecting that of the whiskers on the animal's snout (Ma, 1991). All barrel-related trigeminal nuclei receive input from the whiskers *via* the trigeminal nerve. Evidence accumulated over the past 15 years has demonstrated that at least three distinct axonal pathways project to different regions of the somatosensory thalamic nuclei and from there to the primary and secondary somatosensory (S1 and S2) barrel cortex (Jensen and Killackey, 1987; Deschênes et al., 1996; Pinault and Deschênes, 1998; Pierret et al., 2000; Veinante et al., 2000a; Arnold et al., 2001; Furuta et al., 2009; Wimmer et al., 2010; Oberlaender et al., 2011b; for reviews see Deschênes et al., 1998; Alloway, 2008; Fox, 2008; Deschênes, 2009; Bosman et al., 2011). These pathways have been termed lemniscal, extralemniscal, and paralemniscal pathway and they differ in their brain stem origin, their thalamic relay stations and their neocortical target structures/layers (Table 1); a brief description of them is given below (see also Table 1 and Figure 2).

**Table 1:** **Pathways in the whisker-to-barrel cortex system**.

**Pathway**	**Brainstem (trigeminal nuclei)**	**Thalamus (*contralateral*)**	**Neocortex (main target regions are italicized and bold)**	**Response type**
Lemniscal (1)	N. principalis	VPMdm core barreloid	S1 Layer 3	Single whisker
			*S1 Layer 4 barrels*	
			S1 Layer 5B	
			*S1 Layer 6A*	
Lemniscal (2)	N. principalis	VPMdm head barreloid	*S1 Layer 4 septa*	Single whisker
				Multiple whisker
Extralemniscal	N. interpolaris (caudal)	VPMvl No clear barreloid detectable	S1 Layer 3 dysgranular	Multiple whisker
			S1 Layer 4 dysgranular	
			S1 Layer 6A dysgranular	
			*S2 Layer 4*	
			*S2 Layer 6*	
Paralemniscal	N. interpolaris (rostral)	POm	S1 Layer 1 barrels and septa	Multiple whisker
			*S1 Layer 4 septa*	
			*S1 Layer 5A barrels and septa*	
			S2	

Pathways from the brain stem to the barrel cortex indicating the name of the pathway, the brainstem relay, the thalamic relay station, the target regions in the primary and secondary somatosensory cortices and whether the neuronal response is elicited only by a single or by several (multiple) whiskers.

The ***lemniscal pathway*** relays whisker signals through the principal trigeminal nucleus (PrV) and projects from there to the dorsal medial region of VPM nucleus (VPMdm) of the thalamus. Here, the axons from a specific “barrelette” in the trigeminal nucleus contact neurons in the corresponding contralateral thalamic “barreloid,” a cytoarchitectonic structure which is a curved, tapering rod with an oblique orientation (Hoogland et al., 1987; Land et al., 1995; Haidarliu and Ahissar, 2001; Varga et al., 2002). The lemniscal pathway can be subdivided into two separate branches depending on the target region in the VPM barreloid (Table 1, Figure 2). The so-called “lemniscal (1)” branch innervates the core region of the VPM barreloid while the “lemniscal (2)” branch project to its head region. Axons arising from VPM neurons in the barreloid core innervate predominantly layer 4 and 6A of the corresponding S1 barrel column and to a lesser extent also layer 3 and 5B (Bureau et al., 2006; Cruikshank et al., 2010; Oberlaender et al., 2011b) and have single-whisker receptive fields (Ito, 1988; Simons and Carvell, 1989; Armstrong-James and Callahan, 1991; Diamond et al., 1992; Brecht and Sakmann, 2002b). A small fraction of VPM neurons may have larger receptive fields but their exact location in the barreloid was not determined (Minnery et al., 2003). In marked contrast, afferents arising from the head of the VPM barreloid [i.e., those in the lemniscal (2) branch] innervate exclusively neurons located in the L4 “septa” and have multi-whisker receptive fields (Urbain and Deschênes, 2007; Furuta et al., 2009).

**Figure 1:**
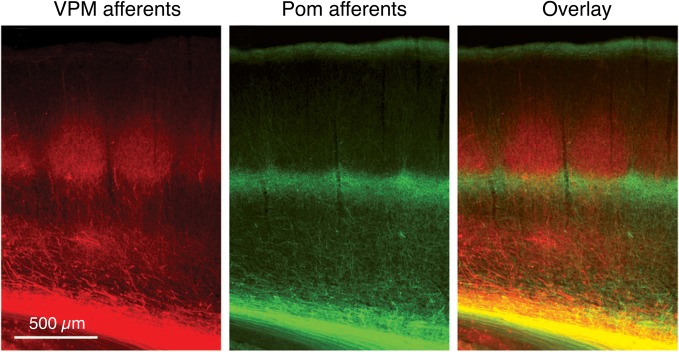
**Dual labeling of axons projecting from the VPM and POm axons.** Labeling of VPM and POm axons in the same animal by adeno-associated virus-mediated expression of different fluorescent proteins. **VPM afferents** (red) in a thalamocortical barrel cortex slice. **POm afferents** (green). **Overlay** of VPM and POm labeled thalamocortical axons illustrating afferent sparse zones of low fluorescence (i.e., low thalamocortical innervation). There is potential overlay of VPM and POm afferents in the deeper portion of barrels (yellow). Modified from Wimmer et al. (2010), with permission of the Society for Neuroscience.

The majority of the lemniscal thalamic afferents, in particular those arising from the core VPM barreloids, show a clear barrel-column related axonal projection with profuse branching at the level of a single barrel in layer 4 (Figures 1, 3A; Jensen and Killackey, 1987; Chmielowska et al., 1989; Lu and Lin, 1993; Pierret et al., 2000; Arnold et al., 2001; Meyer et al., 2010a,b; Oberlaender et al., 2011b). However, some VPM neurons possess axons that bifurcate in layer 6 or 5 to innervate two or more barrel columns; these may arise from other regions, e.g., the barreloid head (Pierret et al., 2000). This structural feature may contribute to the relatively large subthreshold whisker-related receptive fields (as defined by EPSP recordings) in layer 4 that have been observed in *in vivo* studies (Brecht and Sakmann, 2002a). Nevertheless, even these bifurcating thalamocortical axons showed a clear preference for a barrel-related columns (Arnold et al., 2001).

**Figure 2:**
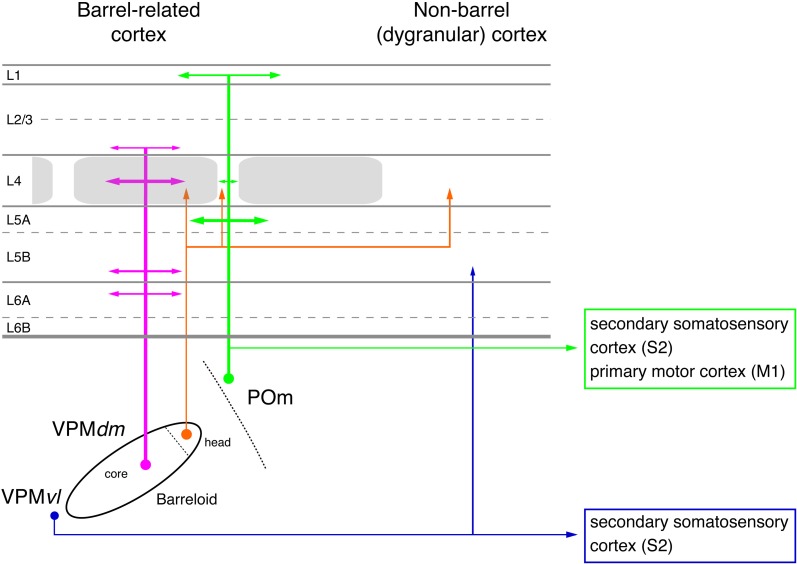
**Pathways from thalamus to the primary somatosensory cortex (S1).** The figure shows the section of the four different whisker-related pathways from the thalamus to the primary somatosensory cortex. The thalamus is represented by a single barreloid in VPM; the border between POm and VPM by a dashed line. The input stations in the brainstem nuclei have been omitted in this diagram. Magenta: lemniscal (1) pathway; orange: lemniscal (2) pathway; green: paralemniscal pathway; blue: extralemniscal pathway. The term “dysgranular cortex” in S1 defines the region in and around the barrel field in which layer 4 shows no clear barrel structure. Abbreviations: VPMvl, ventrolateral portion of a barreloid in the ventroposterior medial nucleus of the thalamus; VPMdm, dorsomedial portion of a barreloid in the VPM.

The ***paralemniscal pathway*** originates from neurons located in the rostral (oral) section of the interpolar spinal trigeminal nucleus (SpV; Veinante et al., 2000a); the SpV lies posterior to the PrV. This section shows no “barrelette”-like subdivisons; neurons in this structure show multi-whisker responses (Erzurumlu and Killackey, 1980; Peschanski, 1984; Williams et al., 1994; Veinante and Deschênes, 1999). Their axons terminate in the POm nucleus of the thalamus (Lavallée et al., 2005) that does not show “barreloid”-like cytoarchitectonic units like the VPM nucleus. From there, the thalamic afferents project to both S1 and S2 whisker-related cortex. In S1 cortex, the main target regions of POm afferents are neurons in layer 1, and 5A and the septum-related but not barrel-related layer 4 neurons (Table 1, Figure 2 and Herkenham, 1980; Chmielowska et al., 1989; Lu and Lin, 1993; Bureau et al., 2006; Wimmer et al., 2010; but see Furuta et al., 2009). Axon fibers from neurons in the anterior part of POm target preferentially layer 5A of S1 while those from neurons in the posterior part were predominantly found in layer 1 (Ohno et al., 2011). The fact that the target regions of the—predominantly lemniscal—VPM afferents and the paralemniscal POm afferents show a largely complementary distribution (Table 1 and Figures 1, 2 and Koralek et al., 1988; Alloway, 2008; Wimmer et al., 2010) has lead to the hypothesis that there are distinct streams of whisker information processing in rodent barrel cortex. In the cortex itself, these streams are represented by barrel and septal circuits, which are involved in sensory analysis (both barrel and septal circuits) and the modulation of whisking behavior (septal circuits only; for details see Alloway et al., 2004; Alloway, 2008; Chakrabarti and Alloway, 2009). However, these two circuits may not be entirely separate. Septal L4 neurons receive also input from the lemniscal (2) pathway (Table 1; see Furuta et al., 2009); neurons in this region are therefore in a position to integrate the lemniscal and paralemniscal streams to the neocortex at a very early stage. Septal neurons are therefore not simply elements of an intracortical “paralemniscal” pathway as previously suggested (Bureau et al., 2006).

Like the paralemniscal pathway, the ***extralemniscal pathway*** also relays signals through the caudal region of the interpolar SpV which shows a “barrelette”-like organization—in contrast to the oral part of the interpolar SpV which is a relay station in the paralemniscal pathway. It reaches the somatosensory cortex through the ventral-lateral region of the VPM (VPMvl), the tail region of the barreloids. In contrast to VPMdm, the VPMvl region of the somatosensory thalamus shows no clear subdivision into barreloids or similar neuron clusters. Extralemniscal afferents target to a moderate degree the dysgranular regions of layers 3, 4, and 6 of S1 barrel cortex and densely neurons in layers 4 and 6 of S2 cortex (Table 1 and Pierret et al., 2000; Bokor et al., 2008). Nevertheless, even here a distinct vertical projection pattern can be observed. It has been suggested that the distinct whisker-to-barrel cortex pathways are associated with specific sensory modalities (Yu et al., 2006). The lemniscal pathway has been associated with a combined whisking-touch signal while the extralemniscal pathway is hypothesized to mediate only the contact signal and the paralemniscal pathway only the sensor motion (whisking) signal.

## Cortical signal processing in the barrel-related column

During the past 10–15 years significant advances in the study of signal processing in a barrel-related cortical column have been made using anterograde or retrograde axonal labeling, paired electrophysiological recording, and connectivity mapping experiments using either caged glutamate release or—more recently—light activation of neurons that are selectively manipulated to selectively express the light-sensitve bacterial ion channel “channelrhodopsin 2” (ChR2; Petreanu et al., 2007; Zhang et al., 2007; Scanziani and Häusser, 2009).

### Input to the neocortex

**Figure 3:**
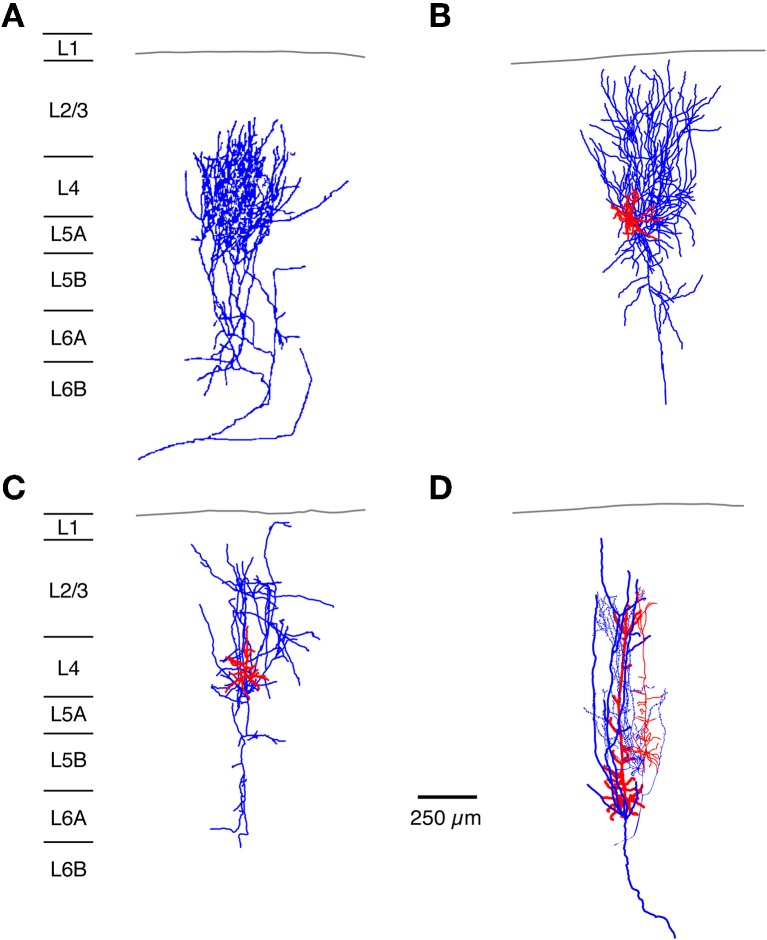
**Neuronal elements in the S1 barrel cortex with a predominantly vertical axonal organization.** The figure shows four types of axonal projections (blue) with a predominantly vertical axonal projection that is largely confined to a barrel column in the whisker-related S1 cortex. **(A)** Most thalamic afferents from VPM nucleus of the thalamus arborize extensively in layer 4 in a barrel-restricted fashion, **(B)** L4 spiny stellate cell, **(C)** L4 star pyramidal cell, **(D)** corticothalamically projecting L6A pyramidal cell. The dendritic domain of intracortical neurons is given in red. (Modified from Oberlaender et al. (2011b) **(A)**, from Feldmeyer et al. (1999) **(B,C)**, from Zhang and Deschênes **(D)** with permission of John Wiley and sons on behalf of The Physiological Society, the Society for Neuroscience and Oxford Journals).

Virtually all cortical layers of the whisker-related S1 cortex receive thalamic input from either the VPM or POm neurons as mentioned above. This input shows a clear vertical organization (Figures 1, 2A). The highest density of thalamocortical axon collaterals can be found in cortical layer 4 (Bernardo and Woolsey, 1987; Jensen and Killackey, 1987; Chmielowska et al., 1989; Senft and Woolsey, 1991; Pierret et al., 2000; Wimmer et al., 2010; Oberlaender et al., 2011b) which can therefore be regarded as the major input layer of the barrel and also of other sensory cortices. As can be seen in Figure 3A, the lemniscal VPM afferents show many bifurcations at the level of a single barrel in layer 4; in addition, lower layer 3 and layers 5B and 6 are also innervated by axons from the VPM neurons; neurons in layer 2 receive only sparse innervation by thalamic axons, while those in layer 6B receive almost none (Meyer et al., 2010a; Oberlaender et al., 2011b). In layer 4 as well as other cortical layers the lemniscal afferents target both excitatory neurons and inhibitory interneurons (Hersch and White, 1981; White et al., 1984; Porter et al., 2001; Beierlein et al., 2003). The majority of VPM afferents form synaptic connection with excitatory neurons because these outnumber L4 interneurons by far. However, they show also a strong convergence onto L4 interneurons (Bruno and Simons, 2002; Cruikshank et al., 2007). Thus, layer 4 is the major and dominant input layer in a barrel-related column in which the bouton density of VPM axons is higher than in any other cortical layers; most of theset boutons form synapses with L4 spiny stellate cells and star pyramidal neurons (Bruno and Sakmann, 2006; Oberlaender et al., 2011b). It should be noted, however, that synaptic contacts established by the thalamocortical afferents are only about 10–20% of the total number of synaptic contacts in layer 4 (White and Rock, 1979; Benshalom and White, 1986) and are therefore considerably outnumbered by intracortical synaptic connections. *In vivo* during anaesthesia, the monosynaptic thalamocortical (VPM-L4) EPSP is about 1 mV in amplitude; during mild sedation this amplitude drops even further, suggesting a relatively low synaptic efficacy. Because synaptic inputs from VPM onto L4 neurons are both relatively frequent and show a high degree of synchronous activity amplification *via* intralaminar L4 synaptic connections is nevertheless not required to drive the intracortical signal flow (Brumberg et al., 1999; Miller et al., 2001; Bruno and Sakmann, 2006).

It has been proposed that barrels in layer 4 can be functionally classified into “mini”-columns containing neurons that are preferentially activated by whisker deflections at a specific angle. Such “angular tuning” domains are formed by convergent synaptic inputs from thalamocortical neurons with corresponding angular preferences. Processing within such domains may depend on local connectivity among vertically aligned barrel neurons (Bruno et al., 2003; Andermann and Moore, 2006; Furuta et al., 2011) which have been reported to be organized in clusters (Feldmeyer et al., 1999; Lübke et al., 2000). In addition, cytoarchitectonic sub-barrel domains have been identified in large but not small barrels in mouse S1 cortex that are enriched in thalamocortical axon terminals (Land and Erickson, 2005). It is tempting to speculate that sub-barrels are the morphological correlates of functional “angular tuning” domains. “Angular tuning” domains similar to those observed at the level of layer 4 have also been confirmed for layer 2/3 in rat whisker-related S1 cortex (Andermann and Moore, 2006; Kremer et al., 2011).

## Intracortical excitation

Within a defined cortical area such as the barrel cortex, neuronal connections can be subdivided into three major groups: local, intralaminar connections, translaminar connections, and connections between cortical columns; in addition there are also long-range synaptic connections that link neurons to other cortical areas and subcortical target regions. The barrel cortex is ideal to investigate the functional and structural properties of such connections because of its clearly visible somatosensory topography, which relates the sensory periphery to the cortical signal processing area (Fox, 2008; Bosman et al., 2011). Because individual synaptic connections have mainly been characterized for the S1 cortex, the review concentrates on this type of connections.

### Layer 4 serves to distribute intracortical excitation

The neuronal targets of thalamocortical afferents in layer 4 are spiny stellate cells, star pyramids, and L4 pyramidal neurons (Brecht and Sakmann, 2002a; Staiger et al., 2004; Bruno and Sakmann, 2006). However, the latter type of neurons has not been identified in other studies (Lübke et al., 2000; Egger et al., 2008). Major functional differences have not been reported for excitatory L4 neurons (Feldmeyer et al., 1999; Lübke et al., 2000; but see Cowan and Stricker, 2004; Staiger et al., 2004). The two different types of excitatory L4 neurons may differ in their synaptic connectivity: Star pyramids have been reported to receive weak and sparse synaptic input from other cortical layers in the home column while spiny stellate cells do not (Schubert et al., 2003).

**Figure 4:**
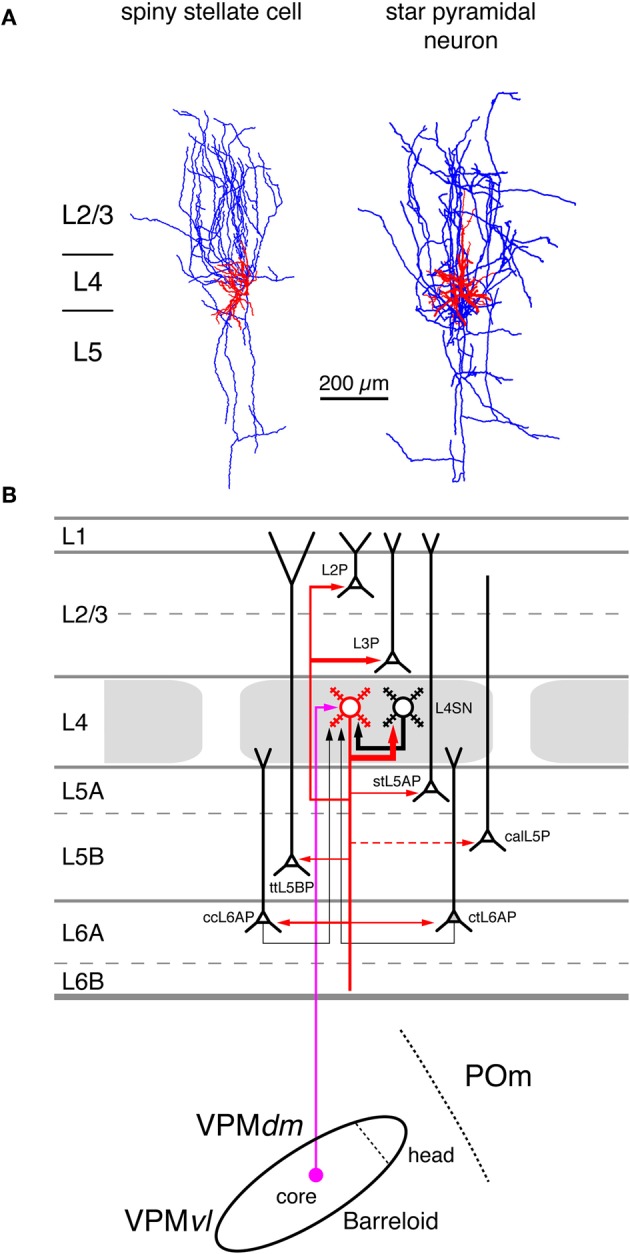
**Excitatory synaptic input–output relationship in layer 4 of the S1 barrel cortex. (A)** Reconstructions of a L4 spiny stellate cell (left) and a L4 star pyramidal neuron (right) in rat barrel cortex (Feldmeyer et al., 1999). Modified with permission of John Wiley and Sons on behalf of The Physiological Society. **(B)** Diagram of the excitatory synaptic connections of and onto L4 spiny neurons (red neuron with blue axon) in the barrel cortex. Although layer 4 contains both spiny stellate and star pyramidal neurons and a few pyramidal cells only spiny stellate cells are shown for simplicity. Note that L4 spiny neurons provide synaptic output to virtually all layers in a barrel column. For detailed information on the location of synaptic contacts and differences in the connectivity of the three different excitatory L4 neurons see text. The thalamic region is represented by a single barreloid in the VPM nucleus of the thalamus; the VPM/POm border is marked by a dashed line. **Red neuron**; Dendrites and axon of the neuron for which the input–output relationship is described in this figure. Different cortical layers as indicated on the left. The thickness of the red arrows pointing to a postsynaptic (black) neurons indicates the connection probability between this and the black neurons as well as cortical and subcortical areas. The dashed red arrow in layer 5 marks a likely but not yet verified synaptic connection onto a corticocallosal L5 pyramidal cell. It should be noted that **Black neurons**: Dendrites and axon of neurons sending to and receiving synaptic input from to the red neuron. The thickness of the black arrows pointing to the red neuron indicates the connection probability between these neurons and the red neuron. **Light blue arrows**: Excitatory synaptic input from cortical regions outside the S1 barrel cortex. **Magenta arrow**: Synaptic input from the VPM (lemniscal (1) pathway. **Green arrow**: Synaptic input from the POm (paralemniscal pathway). However, for L4 spiny neurons synaptic input from outside thhe barrel cortex originates almost exclusively from the core of the barreloid in the dorsomedial part of the VPM. **Abbreviations**: VPM, ventroposterior medial nucleus of the thalamus; dm, dorsomedial part; vl, ventrolateral part; POm, posterior medial nucleus of the thalamus; L2P, L2 pyramidal cell; L3P, L3 pyramidal cell; L4SN, L4 spiny neuron; stL5P, slender-tufted L5A pyramidal cell; ttL5BP, thick-tufted L5B pyramidal cell; calL5P, corticocallosal L5 pyramidal cell; ccL6AP, corticocortical L6A pyramidal cell; ctL6AP, corticothalamic L6A pyramidal cell.

The axonal and dendritic domain of spiny stellate cells and star pyramids show a column-related topology but differ in the fine structure of the dendritic and axonal projections (Lübke et al., 2000; Egger et al., 2008). The dendritic domain of L4 spiny neurons remains largely within a barrel in layer 4 (with the exception of the apical dendrite of star pyramids) while their axonal domain is largely columnar with a very high density of axon collaterals in layers 4 and 2/3 (Figures 3 and 4A and Feldmeyer et al., 1999; Lübke et al., 2000; Brecht and Sakmann, 2002a; Lübke et al., 2003; Bruno and Sakmann, 2006; Egger et al., 2008). This columnar topography is developmentally regulated: during early postnatal stages (postnatal day (P) 4 to 10) the axon projects over the borders of the home barrel column while it is largely confined to it by the end of the third to fourth postnatal week (Bender et al., 2003; Radnikow et al., 2010). In another study such a developmental regulation was not observed; however, this study used a much narrower age range (P8–16; Bureau et al., 2004). Other neurons in the neocortex such as the corticothalamically projecting L6A pyramidal cells show a similar columnar organization of their axon (Figures 3 and 7A; Zhang and Deschênes, 1997; Kumar and Ohana, 2008).

A small fraction of L4 spiny neuron axons projects into neighboring barrel-related columns where they branch profusely in the neighboring barrel but still obey column borders (Egger et al., 2008). These neurons may serve in interbarrel signaling and could enlarge the subthreshold receptive fields of L4 spiny neurons. Furthermore, some L4 star pyramids exhibit long-range projections over several barrel-columns or rows, both in layer 4 and in infragranular layers (Lübke et al., 2000; Brecht and Sakmann, 2002a; Egger et al., 2008).

In rodent barrel cortex, L4 spiny neurons form cell clusters in which they are highly interconnected. Reported connectivity ratios range from 25 to 36% (about 20–30% of which are reciprocal) and are thus the highest reported for excitatory neocortical synapses (Feldmeyer et al., 1999; Petersen and Sakmann, 2000; Lefort et al., 2009). The presynaptic excitatory L4 neuron forms between two and five synaptic contacts on the dendrites of the postsynaptic neuron at an average geometric distance of ~70 μm. Monosynaptic connections between L4 spiny neurons are relatively efficacious (mean unitary EPSP (uEPSP) amplitude: 1.6 mV) and highly reliable, indicative of a high release probability. In a few L4-L4 connections, single uEPSPs were found to be sufficiently large to evoke action potentials (Feldmeyer et al., 1999). The L4-L4 connection is almost the only intracortical synaptic input L4 spiny neurons receive (Figure 4B). The connectivity ratios with excitatory neurons in all other layers of S1 barrel cortex are extremely low, often below 1% (Lefort et al., 2009). However, it is likely that a connections between L4 spiny neurons and L6 pyramidal neurons exist as the the strong axonal arborization of the L6 axon suggests (Pichon et al., 2012).

From layer 4 the incoming thalamocortical excitation spreads to other cortical layers, most prominently to layer 2/3 which shows also a high density of L4 spiny neuron axon collaterals (Lübke et al., 2000, 2003). L2/3 pyramidal cells are strongly innervated by L4 spiny neurons in the home barrel-related column; the connectivity ratio ranges between 10 and 15% (Feldmeyer et al., 2002; Silver et al., 2003; Lefort et al., 2009). The presynaptic L4 axons form synaptic contacts mainly with the basal dendritic arbor of the postsynaptic L2/3 pyramidal cell at an average distance of ~70 μm, i.e., close to the soma. The number of synaptic contacts varies between four and five. Despite their relatively large axonal distance (200–400 μm), synaptic connections between L4 spiny neurons and L2/3 pyramidal cells have a surprisingly high connectivity ratio, are efficacious (uEPSP amplitudes ranging from 0.6 to 1.0 mV) and of a relatively high release probability (*P*_*r*_ ~ 0.8; Feldmeyer et al., 2002; Silver et al., 2003; Sarid et al., 2007; Lefort et al., 2009).

In contrast to the intralaminar L4-L4 connection, L4-L2/3 connections are never reciprocal (Feldmeyer et al., 2002) and connections between presynaptic L2/3 pyramidal cells and L4 spiny neurons are extremely rare (Lefort et al., 2009). In studies on rat neocortex using laser scanning photo-release of caged glutamate in brain slices it has been shown that L4 spiny neurons in a cortical barrel are the most dominant input to barrel-related L2/3 pyramidal neurons (located above a barrel in layer 2/3) while this is not the case for septum-related L2/3 pyramids (i.e., those above a septum in layer 4; Shepherd et al., 2005; Shepherd and Svoboda, 2005). For murine barrel cortex, however, the same group has proposed a different connectivity. Barrel-related pyramidal neurons in lower layer 2/3 (often referred to as layer 3) receive strong input from layer 4 while those in upper layer 2/3 (i.e., layer 2) Septum-related L2 and L3 pyramids, are only weakly innervated by barrel- and septum-related L4 neurons (Bureau et al., 2006). This view is not supported by paired recording studies in both mouse and rat barrel cortex which report comparable connectivity ratios between L4 spiny neurons and pyramidal cells in deep and superficial layer 2/3 (Feldmeyer et al., 2002; Lübke et al., 2003; Lefort et al., 2009). Nevertheless, a decrease in the connectivity with increasing axonal path length is likely because of the reduction in axonal density (and hence the probability of forming synaptic contacts; Lübke et al., 2003).

As discussed above, L4 spiny neurons in a cortical barrel target preferentially other L4 spiny neurons and pyramidal neurons in layer 2/3 of the same barrel-related column. However, they have also been demonstrated to innervate L5A, L5B, and L6A pyramidal cells suggesting the existence of a direct, monosynaptic signal transformation from layer 4 to infragranular layers (in addition to the indirect, disynaptic connection from layer 4 via layer 2/3 to layer 5; see below). The connectivity of L4 spiny neurons with L5A and L5B pyramidal cells is also relatively high with a connectivity ratio of about 10%, but of a lower efficacy (mean uEPSP amplitude of ~0.6 mV) than that of other L4 connections (Schubert et al., 2001, 2006; Feldmeyer et al., 2005; Lefort et al., 2009; Petreanu et al., 2009; Hooks et al., 2011). Synaptic contacts on infragranular neurons are mainly established on the basal dendrites of the L5 pyramidal cells (Markram et al., 1997; Feldmeyer et al., 2005; Petreanu et al., 2009); the distribution of these contacts overlaps to a significant degree with that proposed for thalamocortical synaptic contacts (Petreanu et al., 2009; Meyer et al., 2010a,b; Oberlaender et al., 2011b). In addition to pyramidal cells of layer 5A and 5B, L4 spiny neurons target also pyramidal cells in layer 6, although the observed connectivity ratio was very low (Lefort et al., 2009; Qi and Feldmeyer, 2010; Tanaka et al., 2011). However, synaptic connections between L4 spiny neurons and L6A pyramidal cells exhibit a synaptic target region specificity not found for other L4 connections: L4 spiny stellate cells innervate exclusively the apical tuft of L6A pyramidal cell and show slow EPSPs with rise times exceeding 3 ms. On the other hand, L4 star pyramids target predominantly—but not exclusively—basal and deep apical oblique dendrites of L6A pyramidal cells and give mainly rise to fast EPSPs (Qi and Feldmeyer, 2010).

From the available data it appears that cortical layer 4 acts as a “hub” of intracolumnar information processing because neurons of this layer signal to all other cortical layers in the same barrel-related column with the possible exception of layer 1. Although L4 spiny neurons do not project outside the barrel cortex and are largely confined to a barrel column they are an integral part of many neuronal subnetworks that are involved in both feed-forward signaling within the S1 cortex and to S2 and the primary motor (M1) cortices (*via* L2/3 and corticocortically projecting L5 and L6 pyramidal neurons, see below) and feedback signaling to structures such as the thalamus (*via* corticothalamic L5 and L6 pyramidal neurons, see below).

### Vertical and horizontal spread of synaptic signaling in layer 2/3 of the barrel cortex

**Figure 5:**
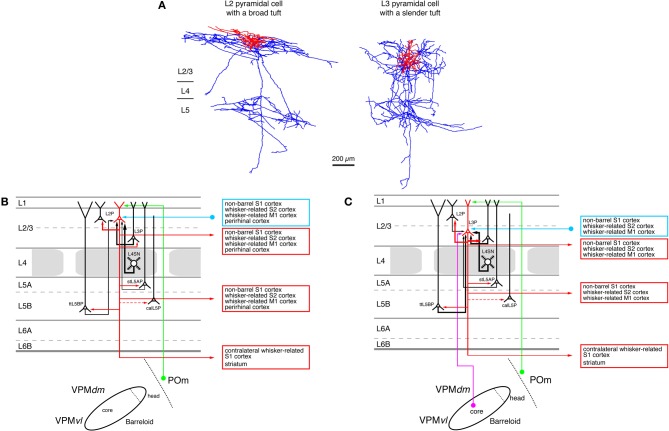
**Excitatory synaptic input–output relationship in layer 2/3 of the S1 barrel cortex. (A)** Reconstructions of a pyramidal cell located in the upper half of layer 2/3 (L2 pyramidal cell, left) and a pyramidal cell located in the lower half of layer 2/3 (L3 pyramidal cell, left) of rat barrel cortex (Bruno et al., 2009); modified with permission of the Society of Neuroscience. Note that the apical tuft of the L2 pyramidal cell is substantially larger than the basal dendritic tree of that neuron while L3 pyramidal cells have slender apical tufts. Modified with permission from the Society of Neuroscience. **(B)** Diagram of the excitatory synaptic connections of and onto L2 pyramidal cells (red neuron with blue axon) in the barrel cortex. Only synaptic input from neurons and regions relevant for L2 pyramidal cells is shown in this graph. For detailed information on the location of synaptic contacts and possible subtypes of L2 pyramidal cells see text. **(C)** Diagram of the excitatory synaptic connections of and onto L3 pyramidal cells (red neuron with blue axon) in the barrel cortex. Only synaptic input from neurons and regions relevant for L3 pyramidal cells is shown in this graph. For detailed information on the location of synaptic contacts and possible subtypes of L3 pyramidal cells see text. Color coding and abbreviations as in Figure 4.

The spread of excitation from the thalamus to layer 4 and from there to layer 2/3 is mostly vertical and largely confined to the barrel-related column (Petersen and Sakmann, 2001; Feldmeyer et al., 2002; Laaris and Keller, 2002; Lübke et al., 2003; Shepherd et al., 2003, 2005; Shepherd and Svoboda, 2005). In addition, L3 pyramidal neurons (Figure 5A, right neuron) also receive (Jensen and Killackey, 1987; Arnold et al., 2001; Meyer et al., 2010b; Oberlaender et al., 2011b) intput from the VPM nucleus of the thalamus (Figure 5C).

L2 Pyramidal cells (Figure 5A, left neuron) have short apical dendrites with relatively large tufts in layer 1 while L3 pyramids have longer apical dendrites with more slender tufts (Lübke et al., 2003; Shepherd and Svoboda, 2005; Feldmeyer et al., 2006; Oberlaender et al., 2011b). It is conceivable that the different types of apical tufts of L2 and L3 pyramids is the structural basis of a differential POm input (*via* layer 1, see Figure 5B) to these neurons: large tufts are in a position to form more synaptic contacts because of the number of dendrites in this layer. However, this has not been demonstrated directly.

Most pyramidal neurons in layer 2/3 have axonal domains that exhibit a “butterfly” appearance: a long stem axon that runs down into the white matter and has several long-range collaterals projecting horizontally mainly in layers 2/3 and 5 over the entire barrel field in S1 and into the ipsilateral S2 and M1 cortex while sparing layer 4 almost completely (Figure 5A; Feldmeyer et al., 2006; Larsen and Callaway, 2006; Bruno et al., 2009; Aronoff et al., 2010, see also below). Apart from these, a few L3 pyramidal cells have been identified that exhibit some collateralization in layer 4 (Larsen and Callaway, 2006; Bruno et al., 2009) and have a much narrower axonal field in supra- and infragranular cortical layers (and a high axonal density in the barrel column). Both types of neurons were found above barrels. All these functional data suggest an heterogeneity in the neuronal make-up of layer 2/3. How this is related to the functional role of this layer remains to be determined.

Studies using photo-release of caged glutamate to stimulate synaptic connections onto L2/3 pyramidal cells (Shepherd et al., 2005; Shepherd and Svoboda, 2005; Schubert et al., 2006) revealed that these neurons show differential excitation pattern depending on their location in relation to the barrel field. In these studies it was found that both L2 and L3 pyramidal neurons above a barrel are strongly excited by the subjacent L4 neurons (see above) while L2 pyramidal neurons above a septum between barrels are more excited by L5A pyramidal neurons. Septal L3 pyramidal are only weakly excited by L5A neurons although the somatic distance between pre- and post-synaptic neurons is shorter for this connection type. Because L4 neurons are the major target neurons of the lemniscal (1) thalamic afferents and L5A pyramidal cells targets of the paralemniscal afferents, the authors suggested that the L4-L2/3 (barrel) pathway and the L5A-L2 (septal) pathway represent intracortical continuations of the lemniscal and paralemniscal pathways, respectively. These two pathways have been suggested to converge in layer 2 because L2 pyramidal cells receive input from L3 pyramidal cells that are targeted by both L4 barrel neurons and VPM (lemniscal) thalamic axons (Figures 3B,C; Shepherd et al., 2005; Shepherd and Svoboda, 2005; Bureau et al., 2006). However, layer 2 is not the only cortical layer where the lemniscal and paralemniscal pathway converge: pyramidal neurons in layer 5A receive anddirect input from POm neurons in the paralemniscal pathway and indirect lemniscal input *via* L4 spiny neurons and L3 pyramidal cells (Feldmeyer et al., 2005; Lefort et al., 2009). In addition, the septa between the barrels in layer 4 are innervated by both lemniscal (2) (Furuta et al., 2009) and paralemniscal afferents (Koralek et al., 1988; Alloway, 2008; Wimmer et al., 2010) and thus constitute a third region in which these pathways converge (see above). Finally, pyramidal neurons in layer 5B receive input from the—largely lemniscal—VPM nucleus of the thalamus (Wimmer et al., 2010; Oberlaender et al., 2011b) but project back to the—paralemniscal—POm were they synapse onto thalamic relay neurons (Hoogland et al., 1987, 1991; Groh et al., 2008). All this data indicates that lemniscal and paralemniscal pathways cross-talk at multiple stations. It is therefore questionable whether separate lemniscal and paralemniscal pathways exist in the neocortex.

When pyramidal cells in layer 2/3 are depolarized above the action potential threshold intracortical signaling spreads *locally* to neighboring L2/3 pyramidal cells (Egger et al., 1999; Holmgren et al., 2003; Feldmeyer et al., 2006; Lefort et al., 2009; Hardingham et al., 2010) and *vertically* to deeper cortical layers (and here mainly to L5A and L5B pyramidal neurons; Reyes and Sakmann, 1999; Lefort et al., 2009; Petreanu et al., 2009; Hardingham et al., 2010; Hooks et al., 2011) but also *horizontally* across several barrel columns both within layer 2/3 and 5 (Adesnik and Scanziani, 2010); L2/3 pyramidal cells are thus in a position to integrate the activity of several columns surrounding their home barrel column.

*Local* synaptic connections between pairs of L2/3 pyramidal cells have a connectivity rate of 10–20%. Their mean uEPSP amplitude is about 0.4–1.0 mV with a release probability (*P*_*r*_ ~ 0.7–0.8), a value comparable to that observed for excitatory L4-L2/3 connections. They establish between two and four synaptic contacts at a mean geometric distance ~90 μm from the postsynaptic L2/3 pyramidal cell soma; the majority of these synaptic contacts can be found on basal dendrites with a few contacts being formed with proximal apical oblique dendrites (Feldmeyer et al., 2006). Synaptic connectivity ratios are, however, far from fixed: For L2/3 pyramids in S1 barrel cortex it has been shown that sensory deprivation affects the local (i.e., L2/3-L2/3 pyramidal cell) connectivity and connection strength (Cheetham et al., 2007). In the deprived region the connectivity is reduced without concomitant changes in synaptic efficacy while in the spared region connections are strengthened with an otherwise unaltered connectivity. This indicates an experience-dependent regulation of connectivity in the neuronal microcircuitry that serves to expand the representation of the spared, sensory active cortex into the deprived regions. Similar mechanisms may work for other cortical connections.

A substantial fraction of L2/3 pyramidal cell axons descend to *deeper* cortical layers where they arborize extensively, in particular in layer 5A and 5B. Here L2/3 pyramidal neurons establish synaptic contacts predominantly with the basal dendrites of L5A and L5B pyramidal neurons (Reyes and Sakmann, 1999; Schubert et al., 2006; Lefort et al., 2009; Petreanu et al., 2009). One study suggests that L2/3 pyramidal cells are more strongly connected to L5 pyramidal cells when they are located above barrel walls (Dodt et al., 2003). Synaptic connections of L2/3 pyramids onto L5 pyramids are of relatively low efficacy (0.1 mV at postnatal day 28) and display short-term facilitation, indicative of a low release probability (Reyes and Sakmann, 1999). L2/3 pyramidal cells connect with a higher probability to subnetworks of interconnected L5 pyramids while L5 pyramids are more likely to integrate inputs from L2/3 pyramids that are not connected (Kampa et al., 2006). Synaptic signaling from different L2/3 subnetworks thus converges onto specific L5 subnetwork thereby integrating different streams of sensory input.

Besides the vertical signal transformation within the home column of the pyramidal neurons, axons collaterals of L2/3 pyramidal cells expand also substantially *horizontally* in particular within layer 2/3 and 5 to contact surrounding cortical domains (“barrel”-related columns). They are therefore in a position to coordinate synaptic activity in their home column with respect to the neighboring cortical columns in the same cortical hemisphere. Furthermore, L2/3 pyramidal cells are also connected to neurons in the contralateral whisker-related S1 cortex *via* the corpus callosum (White and Czeiger, 1991; Petreanu et al., 2007) and may thus integrate the activity of the two cortical hemispheres. In a separate section below I will describe the structural and functional properties of long-range intracortical connections.

### Layer 5 as the main cortical output layer

**Figure 6:**
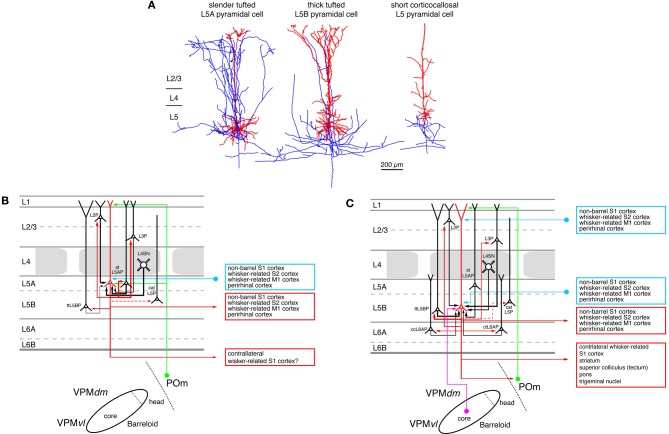
**Excitatory synaptic input–output relationship in layer 5 of the S1 barrel cortex. (A)** Reconstructions of three types of pyramidal cells in layer 5 of the barrel cortex. Slender-tufted pyramidal cells (left) are predominantly located in sublamina 5A (Feldmeyer et al., 2005) while thick-tufted pyramidal cells (middle) are mostly found in sublamina 5B (Lübke and Feldmeyer, 2007). Corticocallosal L5 pyramidal cells (right) are found throughout layer 5. They are characterized by a very diminutive or even absent apical tuft (Le Bé et al., 2007). Modified with permission of the Society of Neuroscience, Springer and Oxford Journals, respectively. **(B)** Diagram of the excitatory synaptic connections of and onto slender-tufted L5A pyramidal cells (red neuron with blue axon) in the barrel cortex. Only synaptic input from neurons and regions relevant for slender-tufted L5A pyramidal cells is shown in this graph. For detailed information on the location of synaptic contacts and possible subtypes of slender-tufted L5A pyramidal cells see text. **(C)** Diagram of the excitatory synaptic connections of and onto thick-tufted L5B pyramidal cells (red neuron with blue axon) in the barrel cortex. Only synaptic input from neurons and regions relevant for thick-tufted L5B pyramidal cells is shown in this graph. Note that thick-tufted L5B pyramidal cells receive synaptic input from virtually all cortical layers. For detailed information on the location of synaptic contacts and possible subtypes of thick-tufted L5B pyramidal cells see text. Color coding and abbreviations as in Figure 4.

Similar to other sensory cortices, layer 5 is the main output layer of the whisker-related S1 cortex. It contains at least two, possibly three main excitatory cell types. These are pyramidal neurons with a slender apical tuft with only few axonal collaterals in layer 1 (Figure 6A, left neuron), those with apical dendrites exhibiting thick, elaborate terminal tufts (Figure 6A, middle neuron) or those that have only short, virtually untufted apical dendrites (Figure 6A, right neuron). The majority of the slender-tufted pyramidal neurons is located in sublayer 5A (Manns et al., 2004; Feldmeyer et al., 2005; Schubert et al., 2006; de Kock et al., 2007; Hooks et al., 2011; Oberlaender et al., 2011b), although some of them are also present in sublayer 5B. In contrast, thick-tufted pyramidal cells are mainly found in sublamina B of layer 5 where also most of the untufted pyramidal cells are found (Larsen and Callaway, 2006). Both slender- and thick-tufted pyramidal cells in layer 5 have been shown to receive synaptic input from the thalamus (Petreanu et al., 2009; Meyer et al., 2010b; Oberlaender et al., 2011b). The slender-tufted L5A neurons receive afferents from the POm nucleus of the thalamus on their basal dendrites and apical tufts (Figure 6B). The thick-tufted L5B pyramidal cells receive VPM thalamic afferents also predominantly on the basal dendrites; however, a few synaptic contacts are also formed with the apical oblique and the terminal tuft dendrites (Figure 6C). Thus, synaptic inputs from the somatosensory thalamic nuclei to L5 pyramidal neurons largely overlap with their main intracortical synaptic inputs from layers 4 (in the case of the L5A pyramids) and 2/3 (in the case of the L5B pyramids).

Short L5 pyramidal cells have extensive axonal projections predominantly to super-granular layers 2/3, in particular to the deeper portion sof this layer. In layer 5 the axon density is considerably lower (not shown in Figure 6A but see Larsen and Callaway, 2006). At least a subset of the short L5 pyramidal cell has axonal projections via the corpus callosum to the contralateral (S1) cortex (Larsen et al., 2007; Le Bé et al., 2007) like the short L5 pyramids in visual and auditory cortex (e.g., Games and Winer, 1988; Hübener and Bolz, 1988; Hübener et al., 1990; Koester and O'Leary, 1992).

Slender-tufted L5A pyramidal cells possess characteristic extensive and dense axons with many ascending collaterals that innervate predominantly the supragranular layers 2/3. Here the axon collaterals cover a wide area of the barrel field and project both within and outside the home barrel column, in fashion reminiscent of short L5 pyramids (Shepherd et al., 2005; Larsen and Callaway, 2006; Hattox and Nelson, 2007; Larsen et al., 2007; Oberlaender et al., 2011a). The infragranular portion of their axon is significantly less elaborate. *In vivo* labeling of these neurons shows that their total intracortical axonal length exceeds that of thick-tufted pyramidal cells in sublamina B of layer 5 by more than a factor of two (Oberlaender et al., 2011a) with a substantial fraction projecting outside the barrel cortex proper. Slender-tufted L5A pyramids project also to ipsilateral cortical areas such as the whisker-related M1 cortex (Mao et al., 2011) and, like the short L5 pyramids, to the contralateral S1 cortex (Figure 6B; Larsen et al., 2007).

The majority of the intracortical axonal collaterals of thick-tufted L5B pyramidal cells (~60%) resides in layer 5; the fraction of supragranular axonal collaterals is markedly lower than that of the slender-tufted or untufted L5 pyramids. Thick-tufted L5 pyramidal cells project to various subcortical target regions such as the thalamic nuclei, the superior colliculus (the tectum), the striatum, and the trigeminal nuclei (Figure 6C; Veinante et al., 2000b; Kozloski et al., 2001; Larsen et al., 2007; Brown and Hestrin, 2009b; Mao et al., 2011). Based on these different target regions further subtypes of thick-tufted (L5B) pyramidal neurons have been proposed (e.g., Hattox and Nelson, 2007; Brown and Hestrin, 2009b; for a review see Brown and Hestrin, 2009a). These L5B pyramid subtypes differ in their passive electrical membrane and their action potential firing characteristics as has been shown for mouse S1 cortex (Hattox and Nelson, 2007); they are therefore likely to process incoming synaptic activity differently. Gene and protein expression profiles can be used for further classification of L5 pyramidal neurons. Studies in recent years have revealed a large degree of diversity in these features (Hevner et al., 2003; Molnár and Cheung, 2006; Nelson et al., 2006; Chen et al., 2008; Groh et al., 2010). Different types of L5 pyramidal cells may form distinct, synaptically connected neuronal subnetworks. For example, Brown and Hestrin (Brown and Hestrin, 2009b) were able to demonstrate that in visual cortex the frequency of monosynaptic connections among corticostriatal L5 pyramidal cells is with 18% significantly higher than among corticocortical or corticotectal pyramids (for which the authors report values of 5 and 7%, respectively). Similar differences were also observed for heterologous L5 pyramidal cell pairs, i.e., of which pre- and post-synaptic neuron belonged to different subclasses (Brown and Hestrin, 2009b). A comparable functional and structural differentiation of L5 pyramidal neurons based on the axonal projection targets has also been observed for rat frontal cortex (Morishima and Kawaguchi, 2006; Morishima et al., 2011; Otsuka and Kawaguchi, 2011) and mouse motor cortex (Anderson et al., 2010). Here, it was found that the synaptic connectivity was higher between neurons with the same subcortical target region (homologous neuron types) than between those with different target regions.

The fact that the probability of finding a synaptic connection as well as its functional properties depend on the identity of both the presynaptic and postsynaptic L5 pyramidal cells support the idea that different neuronal subnetworks exist also in the whisker-related S1 cortex. The local, intralaminar connectivity of slender-tufted (L5A) pyramidal neurons is ~20%, 15% of which are reciprocal connections. Cell bodies and apical dendrites of connected L5A pyramidal cells were located at the border between barrel and septal columns with a clear tendency toward vertical clustering (Frick et al., 2008). Such an organization is consistent with the concept that slender-tufted L5A pyramids belong to a “septal processing” system. This system is recruited by paralemniscal thalamic input from POm and may be involved in the modulation of whisking behavior (Alloway, 2008). Between one and six synaptic contacts are formed between L5A pyramidal cell pairs, mainly on the basal dendrites. These contacts had a low failure rate and an average uEPSP amplitude of 0.3–0.6 mV (Frick et al., 2008; Lefort et al., 2009).

Data from *in vitro* paired recordings demonstrated that L5A pyramidal neurons are more frequently connected to pyramidal neurons in layer 2 and 3 (connectivity ratio 2–4%, respectively; Lefort et al., 2009) than the thick-tufted (L5B) pyramidal neurons (connectivity ratio 1–2%, respectively; Lefort et al., 2009), a finding that is in agreement with the higher axonal density in layer 2/3 found for these neurons (Oberlaender et al., 2011a). This predominant innervation of more superficial L2/3 pyramidal neurons has also been revealed in studies using laser-scanning photo-stimulation by glutamate uncaging or ChR2 activation (Shepherd et al., 2005; Shepherd and Svoboda, 2005; Bureau et al., 2006; Petreanu et al., 2009) although some of these studies note a preferential innervation of L2 pyramidal neurons above the barrel septa.

Figure 6C shows the input output relationship of thick-tufted (L5B) pyramidal neurons Their local connection probability is with 5–20% relatively high (depending on the distance between neuronal cell bodies) with some showing reciprocal connectivity; (Markram et al., 1997; Reyes and Sakmann, 1999; Le Bé et al., 2007; Lefort et al., 2009; Perin et al., 2011). Connections tend to cluster and are thus highly non-random (Song et al., 2005). The L5B-L5B connection probability is lower than that of L5A pyramidal cell pairs but the number of synaptic contacts is larger: between 4 and 8 contacts are established on both basal and apical oblique dendrites at an average geometric distance of 150 μm. In both rat and mouse, L5B-L5B connections are also quite efficacious with reported mean uEPSP amplitudes of 0.7–1.3 mV (Markram et al., 1997; Le Bé et al., 2007; Lefort et al., 2009). While the thick-tufted (L5B) pyramidal cells are to some degree innervated by descending axon collaterals of L5A pyramidal cells, ascending connections from layer 5B to 5A appear to be rare, suggesting a directed signal flow between the two sublaminae (Lefort et al., 2009).

L5A and L5B pyramidal neurons may interact in the following way according to a hypothesis put forward by Oberlaender and coworkers (Oberlaender et al., 2011a): Slender-tufted (L5A) pyramidal neurons have been shown to carry information on the motion and phase of the vibrissae during active whisking but show little if any action potential firing activity after passive touch (Curtis and Kleinfeld, 2009; de Kock and Sakmann, 2009). With their long-range collaterals the slender-tufted (L5A) pyramidal neurons may integrate the barrel-column activity and phase-lock the membrane potential in the dendrites of L2/3 pyramidal neurons to the whisking cycle through their dense axonal collaterals in this layer. They will also recruit thick-tufted pyramidal neurons but to a significantly lesser degree. In contrast, it has been demonstrated that thick-tufted (L5B) pyramidal cells reliably increase action potential firing after *passive* whisker touch (de Kock et al., 2007), possibly through direct synaptic input *via* the VPM thalamic afferents (Bureau et al., 2006; Yu et al., 2006; Petreanu et al., 2009; Meyer et al., 2010a,b; Oberlaender et al., 2011b). In addition, when the slender-tufter (L5A) pyramidal cells and the VPM afferents are activated almost simultaneously during exploratory (sensory-motor) behavior such as during object location, the thick-tufted (L5B) pyramidal cells may be depolarized at both basal dendrites (*via* the VPM afferents) and the apical dendritic tuft (*via* the extensive axon collaterals of the slender-tufted neurons and possibly also *via* afferents from the POm running through layer 1). This will then result in increased neuronal firing, which is subsequently conveyed to other intracortical but also to other subcortical targets.

A subset of thick-tufted L5B pyramidal neurons—which have already been introduced above—receives thalamic input from the VPM and projects back to the POm nucleus of the thalamus (see also the section on long-range connection below). These connections, which may be the cortical relay of a thalamocorticothalamic feedback loop, have been characterized in more detail: Thalamocortically projecting L5B pyramidal cells form giant large diameter (2–8 μm) presynaptic terminals and establish glutamatergic synapses (containing Ca^2+^-permeable AMPA and NMDA receptors) with POm relay neurons (Hoogland et al., 1987, 1991; Bourassa et al., 1995; Groh et al., 2008; Liao et al., 2010). However, it has also ben hypothesized that this connection may be an integral element of a sequential, feed-forward signal transfer from VPM via layer 5B in S1 cortex to POm and from there to other S1 laminae and other cortical areas (i.e., a transthalamic signaling process) such as the S2 or the M1 cortex [see.e.g., (Killackey and Sherman, 2003; Guillery and Sherman, 2011)]. However, this point remains still an open question. It is also possible that L5B pyramidal cells are elements in both the feedforward and the feed-back pathways described above.

The L5B-POm synapses have a high release probability (*P*_*r*_ ~ 0.8) and are sufficiently strong to elicit multiple action potentials in the POm neurons. However, spontaneous *in vivo* activity of the L5B pyramids counteracts this “driving” action through a strong short-term synaptic depression and hence results in a depression of action potential transfer. The L5B-POm giant synapse may therefore have two modes of action: During *high* spontaneous activity, the synapse is suppressed and only synchronous activity of several inputs—possibly arising from multi-whisker deflections—will cause the postsynaptic POm neuron to spike: the synapse acts as a coincidence detector. In contrast, when the spontaneous activity is *low*—e.g., during active whisking or cortical silence—a single, asynchronous input will result in the firing of the POm neuron. Thus, depending on the rate of spontaneous activity, the L5B-POm giant synapse operates either as a detector of neuronal synchrony or cortical silence (Groh et al., 2008).

Synaptic connections between short, corticocallosally projecting L5 pyramidal neurons (Figure 6A, right neuron) in somatosensory cortex are quite distinct from those between other L5 pyramidal neuron types in a number of features (Le Bé et al., 2007). Their connectivity ratio was with 3% considerably lower than for the other types of L5 pyramidal neuron. The release probability at this synaptic connection was with 0.4 also exceptionally low; however, the average uEPSP amplitude was comparable to that of other pyramidal neuron connections in the barrel cortex. Corticocallosally projecting L5 pyramidal neuron pairs formed between one and six synaptic contacts, mainly on the basal dendritic tree at an average distance of ~130 μm. The likely connections (based on the axonal projection pattern; see above and Larsen and Callaway, 2006) between short L5 pyramidal cells and those in layer 2/3 (based on the axon projection pattern of the short L5 have not yet been characterized).

Figures 6B and C summarize what is presently known about the intra- and extracortical connectivity pattern of slender- and thick-tufted pyramidal cells. It should be noted that thick-tufted L5B pyramidal cells receive synaptic input from virtually all cortical layers and project to numerous intra- and subcortical target regions. However, the schematic diagram shown here cannot cover the emerging structural and functional diversity as well as the differential connectivity of L5B pyramidal cells (Brown and Hestrin, 2009a,b). Therefore, this picture is likely to change in the near future.

### The role of cortical layer 6

**Figure 7:**
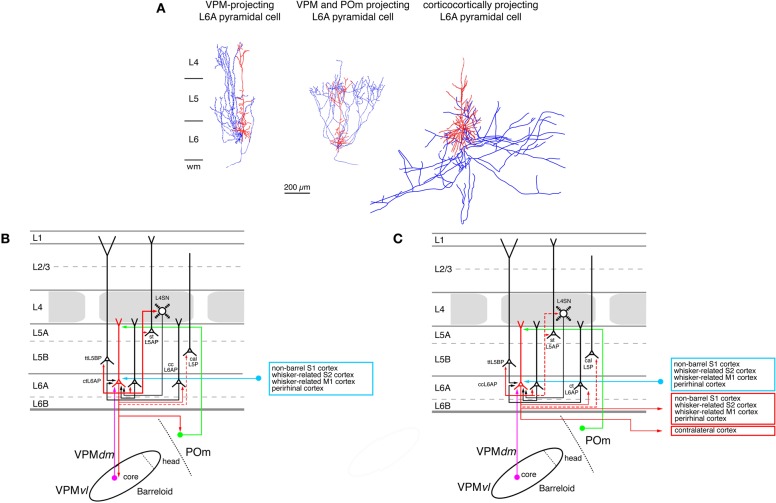
**Excitatory synaptic input–output relationship in layer 6 of the S1 barrel cortex. (A)** Reconstructions of three types of pyramidal cells in sublamina A of layer 6 in the rat barrel cortex (Zhang and Deschênes, 1997); modiefied with permission of the Society for Neuroscience. L6A pyramidal cell projecting exclusively back to the VPM nucleus of the thalamus (left), L6A pyramidal neuron projecting to both the VPM and the POm nuclei of the somatosensory thalamus (middle) and a corticocortical L6A pyramidal cell. The apical trees of L6A pyramidal cells terminate between upper layer 5 and lower layer 3 and have very small or even no tuft. Modified with permission from the Society for Neuroscience. **(B)** Diagram of the excitatory synaptic connections of and onto corticothalamic L6A pyramidal cells (red neuron with blue axon) in the barrel cortex. Only synaptic input from neurons and regions relevant for corticothalamic L6A pyramidal cells is shown in this graph. For detailed information on the location of synaptic contacts and possible subtypes of corticothalamic L6A pyramidal cells see text. **(C)** Diagram of the excitatory synaptic connections of and onto corticocortical L6A pyramidal cells (red neuron with blue axon) in the barrel cortex. Only synaptic input from neurons and regions relevant for corticocortical L6A pyramidal cells is shown in this graph. For detailed information on the location of synaptic contacts and possible subtypes of corticocortical L6A pyramidal cells see text. Color coding and abbreviations as in Figure 4.

Throughout the neocortex layer 6A has been proposed to be the preeminent source of corticothalamic projections (Jones, 1984; Deschênes et al., 1998; Douglas et al., 2004; Sherman, 2005; Shipp, 2007; Fox, 2008; Thomson, 2010). In sensory cortices, corticothalamic projections are generally considered to be elements of a feed-back loop that modulates the response of thalamic relay neurons to peripheral stimuli. In the somatosensory cortex of rodents the relative thickness of infragranular layers in rodents is significantly larger than that of supragranular layers; the thickness of layer 6 is almost equal to that of layer 5 (Ren et al., 1992; Hutsler et al., 2005). The structure of layer 6 reflects its mixed origin with sublamina 6A being derived from the cortical plate (like layer 2–5) while sublamina 6B is a heterogeneous layer that contains neurons that have developed—at least to some extent—from the subplate but may also hold neurons that have migrated there from the cortical plate (Marín-Padilla, 1978). Sublamina 6B contains several distinct types of neurons with highly diverse dendritic domains; their functional role and even their synaptic connectivity has only received little attention to date (Tömböl et al., 1975; Tömböl, 1984; Miller, 1988; Bueno-Lopez et al., 1991; Chen et al., 2009). In contrast, sublamina 6A contains mainly pyramidal neurons with vertically oriented, untufted or sparsely tufted apical dendrites that terminate in lower layer 3–5A. Furthermore, a few neurons with inverted or obliquely oriented dendrites have also been described (Zhang and Deschênes, 1997; Chen et al., 2009). Like L5 pyramidal cells those in layer 6A can be subdivided into at least two different groups with respect to their axonal projection pattern: both groups are equally large groups and consist of pyramidal neurons the axons of which project either predominantly intracortically or corticothalamically to the somatosensory thalamus (Figure 7; Zhang and Deschênes, 1997; Groh et al., 2008; Kumar and Ohana, 2008; Oberlaender et al., 2011b; Tanaka et al., 2011; Pichon et al., 2012); a small group of local circuit neurons (which comprises about 10% of the excitatory L6A neurons) may also exist.

A further subdivision of L6 pyramidal cells can be made on the basis of their axonal projection targets. L6A pyramids that project exclusively to the VPM nucleus of the thalamus have intracortical axon collaterals that show a columnar distribution with most of the collaterals ascending to layer 4 of the home barrel column where they terminate (Figure 7A, left neuron; Zhang and Deschênes, 1997; Kumar and Ohana, 2008). The majority of these neurons are located in the upper to middle portion of sublamina 6A (Bourassa et al., 1995; Killackey and Sherman, 2003). L6A pyramidal cells that target both neurons in the VPM and the POm nucleus have also been identified by Zhang and Dêschenes (Figure 7A, middle neuron; Zhang and Deschênes, 1997) A recent study (Pichon et al., 2012) demonstrated that a subset of L6A pyramidal cells has an extensive axonal domain with many ascending collaterals terminating largely in layer 4 but also in layer 5. Their axon collaterals innervate several barrels ramifying profusely. These neurons may correspond to the corticothalamic, both VPM and POm targeting L6A pyramidal cells. Only few corticothalamic L6A pyramids that target exclusively POm have been described today; these are located in the lower portion of sublamina 6A (Bourassa et al., 1995; Zhang and Deschênes, 1997) and possess relatively short apical dendrites terminating in layer 5. Their intracortical axon collaterals reside mainly in layer 6 with a few branches reaching into lower layer 5B (Zhang and Deschênes, 1997). In addition, another group of L6A neurons has been found in both whisker-related S1 cortex (as well as in S2) that targets the VPMvl, the origin of the extralemniscal pathway (Bokor et al., 2008). The functional role of these corticothalamic L6A neurons may be to provide direct (through layer 6) and indirect (though input from layer 4 and 5B, see below) feed-back modulation of the thalamic activity in the different thalamocortical pathways. They may also be involved in feedforward signaling from the S1 cortex to S2 or M1 cortex.

In contrast to corticothalamic L6A pyramidal neurons, the axons of corticocortical L6A pyramidal cells remain mainly within layer 5 and 6 of the S1 cortex (Figure 7A, right neuron; Zhang and Deschênes, 1997). They project over many barrel columns thus mediating transcolumnar interactions in the infragranular layers of the barrel cortex. The majority of their axonal branches remain within the S1 cortex with some long-range collaterals projecting to the S2 and/or M1 cortex (Figure 7C); they have no obvious subcortical target. It has been suggested that different subtypes of corticocortical L6A pyramids exist, which can be differentiated on the basis of their dendritic and axonal arborization but the exact functional roles of these neurons remain unclear at present (Zhang and Deschênes, 1997; Kumar and Ohana, 2008; Pichon et al., 2012).

Only few studies on the intracortical connectivity of L6A pyramidal neurons in the barrel cortex are currently available and virtually nothing is known about synaptic connections between L6B excitatory neurons. The knowm input–output relationships of corticothalamic and corticocortical L6A pyramids are shown in Figures 7A and B. L6A pyramidal neurons receive input from L5A, L5B, and L6A pyramidal neurons. Homologous L6A connections (i.e., pairs between two corticocortical or corticothalamic L6A neurons) are more frequent than heterologous ones. Both corticocortical and corticothalamic L6 neurons are also presynaptic to L5B pyramids (Mercer et al., 2005; Lefort et al., 2009; Hooks et al., 2011; Tanaka et al., 2011). Corticothalamic L6A pyramidal cells receive a strong and focussed excitatory synaptic input from L4 neurons in their home column, indicating that neurons in this layer are involved in shaping the cortical modulation of the activity in the somatosensory thalamus (Tanaka et al., 2011). Connections with layer 4 were also observed for postsynaptic corticocortical L6A neurones (Qi and Feldmeyer, 2010). Inputs from layer 4 show a distinct sender neuron specificity: spiny stellate neurons contact exclusively the apical tufts of both types of L6A pyramidal cells, while star pyramidal neurons target predominantly the basal dendritic domain and deep apical oblique dendrites This suggests different computational roles for the two types of L4 excitatory neurons in the L4-L6A excitatory synaptic pathway.

Excitatory neurons in sublamina 6B of the barrel cortex have very heterogeneous dendritic morphologies, ranging from short, untufted pyramids with apical dendrites that terminate in layer 5, those with atypically oriented (oblique, horizontal or inverted) “apical” dendrites to multipolar neurons without a clear apical dendrite (Andjelic et al., 2009; Chen et al., 2009). This is in accordance with the description of L6B neurons located in other cortical areas (Tömböl et al., 1975; Tömböl, 1984; Clancy and Cauller, 1999). Few if any studies are available describing the axonal projection pattern of identified L6B neurons. For S1 barrel cortex it has been shown that—like L5B pyramids (see above)—L6B neurons located in both barrel and non-barrel (i.e., septal) cortex innervate POm (Killackey and Sherman, 2003). Furthermore, at least a subset of L6B neurons send also axons to layer 1 [(Mitchell and Cauller, 2001); in this paper layer 6B was termed layer 7]. Future studies are needed to elucidate the morphological and functional properties of the distinct excitatory L6B neuron types.

## Long-range connections within the S1 barrel cortex and to other cortical and subcortical regions

As already indicated in the preceding paragraphs excitatory pyramidal neurons of the rodent barrel cortex project to distant cortical and subcortical target regions and receive afferent input from them. In a recent review, the overall connectivity with these target regions has been described in detail (Bosman et al., 2011). Therefore, the main focus here will be on connections from identified neurons in S1 barrel cortex to other cortical and subcortical regions and their possible function.

Long-range axonal projections were first investigated by anterograde and retrograde transport using classical tracer substances and more recently by viral vectors coupled to fluorescent markers (which can also be used as anatomical tracers). In addition, studies using electrophysiological or optical stimulation (i.e., photo-activation of caged glutamate or ChR2) and optical recording (calcium or voltage-sensitive dye imaging) have revealed the functional synaptic connectivity between these brain regions, which exists often in loops between brain regions.

Within the S1 cortex, L2/3, L5 and a subset of L6 pyramidal neurons in the barrel field possess long-range horizontal axon collaterals. These projections run predominantly along the barrel rows and less along the barrel arcs (Bruno et al., 2009; Adesnik and Scanziani, 2010), i.e., they show a certain direction preference. Long-range projections in S1 cortex may play a role in the modulation of the home column activity with respect to its neighbors. For example, activation of L2/3 pyramidal neurons (Adesnik and Scanziani, 2010) generated rhythmic oscillation in the activity of L2/3 and L5 excitatory (and inhibitory) neurons in the gamma frequency range (~40 Hz) in the home and adjacent barrel columns. On the cellular level, activation of L2/3 pyramids resulted in a lateral suppression of spiking in L2/3 pyramidal cells of neighboring barrel columns but feedforward facilitation of action potential firing in L5 pyramidal cells with very similar spatial profiles. Since layer 2/3 provides the dominant input to layer 5 and this layer is the prominent cortical output, (see above) L2/3 pyramidal cells can drive the output to neighboring barrel-related columns *via* the L5 axons while inhibiting their inputs by depressing L2/3 neuronal activity. In consequence, this coordinated modulation of L2/3 and L5 neurons may result in the lateral expansion of the activity of the principal barrel-related column. Thus, at a given point in time the most active barrel-related column may dominate the output of the S1 cortex at the expense of the adjacent, less active cortical domains.

In addition to the aforementioned horizontal axon collaterals *within* the S1 cortex, L2/3, L5, and L6 neurons project also outside S1. The most prominent connections between whisker-related cortical areas have been reported for S1 and S2 cortex and S1 and M1 cortex; in addition connections to the insular and the perirhinal cortex have been identified (White and DeAmicis, 1977; Welker et al., 1988; Fabri and Burton, 1991; Cauller et al., 1998; Zhang and Deschênes, 1998; Hoffer et al., 2003; Aronoff et al., 2010).

Axonal projections from S1 barrel cortex to the ipsilateral S2 cortex are topographic. The connections between the two whisker-related cortical areas are strong and reciprocal (Carvell and Simons, 1987; Aronoff et al., 2010) and emanate from pyramidal neurons in layers 2/3, 5 (mostly sublamina A) and (to a lesser degree) 6 from neurons in both the barrel and septal columns of the S1 cortex (Koralek et al., 1990; Zhang and Deschênes, 1998; Kim and Ebner, 1999; Chakrabarti and Alloway, 2006). The general connectivity pattern in both cortices is rather similar [see (Hooks et al., 2011) for details] despite some minor differences. Sensory processing in the S2 cortex is likely to be parallel to that in the S1 cortex because whisker signals reach S2 *via* the extralemniscal pathway through the ventrolateral section of the VPM nucleus (Pierret et al., 2000; Bokor et al., 2008) and the POm nucleus (Carvell and Simons, 1987; Spreafico et al., 1987; Alloway et al., 2000; Theyel et al., 2010). This suggests that synaptic input to S2 occurs at virtually the same time as it does in S1.

Because somatosensation in rodents depends on the active movement of their whiskers and the deflection from the free whisker trajectory by an object, an interaction between motor and somatosensory cortex is important not only for object location and recognition but also to modulate and coordinate the whisker movement. Monosynaptic connections between the ipsilateral, whisker-related S1, and M1 cortices have been identified structurally and functionally (White and DeAmicis, 1977; Porter and White, 1983; Miyashita et al., 1994; Izraeli and Porter, 1995; Farkas et al., 1999; Veinante and Deschênes, 2003; Chakrabarti and Alloway, 2006; Ferezou et al., 2007; Petreanu et al., 2009; Aronoff et al., 2010; Sato and Svoboda, 2010; Mao et al., 2011) and are also somatotopically arranged. These projections from the whisker-related S1 to M1 motor cortex arise from a subset of L2/3 and L5A pyramidal neurons in S1 barrel cortex, run through both deep and superficial cortical layers and target L2/3 and L5A neurons in M1 cortex (Porter and White, 1983; Koralek et al., 1990; Miyashita et al., 1994; Aronoff et al., 2010; Sato and Svoboda, 2010; Mao et al., 2011); the majority of these neurons have been suggested to originate in septal columns (Crandall et al., 1986; Alloway et al., 2004; Chakrabarti et al., 2008) and are as such elements of the septal circuits that are hypothesized to modulate whisker motion (Alloway, 2008). M1 neurons receiving input from S1 project directly back to this region thus forming a strong feedback loop. In addition, a small percentage of L6 neurons in S1 also projects to M1 (Mao et al., 2011).

Conversely, ipsilateral M1-to-S1 connections innervate L2/3 and L5 pyramidal neurons *via* axon collaterals that ramify in both layer 5 and 6 as well as layer 1 (Cauller et al., 1998; Veinante and Deschênes, 2003; Petreanu et al., 2009; Matyas et al., 2010; Mao et al., 2011). Specifically, it has been demonstrated that connection from layer 1 in the M1 cortex innervates the apical tufts of L2/3 and L5 pyramidal cells in S1 cortex (Cauller and Connors, 1994; Larkum et al., 1999; Larkum and Zhu, 2002; Zhu and Zhu, 2004; Rubio-Garrido et al., 2009). These neurons receive also direct input from the POm neurons via layer 1 (see above; Wimmer et al., 2010; Ohno et al., 2011) POm neurons are believed to code signals related to whisker position (Yu et al., 2006) while the whisker-related M1 cortex is involved in the voluntary whisker control (Berg and Kleinfeld, 2003). It is therefore conceivable that synapses in layer 1 established between POm and M1 axons and the apical tuft of L2/3 and L5 pyramidal cells serve to integrate signals related to whisker movement and position. This may involve the activation of Ca^2+^ action potential in the apical tufts of the pyramidal cells (Larkum et al., 1999; Larkum and Zhu, 2002).

Finally, the barrel-related M1 cortices in the two brain hemispheres are interconnected *via* the claustrum (Smith and Alloway, 2010) and L6 pyramidal cells in this cortex project back to the contralateral ventromedial and the ventrolateral nuclei of the thalamus (Alloway et al., 2008), suggesting a modulatory role of M1 cortex in S1 signaling and in the bilateral coordination of whisker movement.

In addition to their role in sensory perception, it has recently been shown that M1 and S1 cortex have different and independent roles in whisker motion (Matyas et al., 2010). While M1 drives whisker protraction *via* the brainstem reticular formation and the facial nucleus, the S1 cortex induces the retraction of the whisker *via* the SpV trigeminal nucleus and also the facial nucleus. This finding argues for a strong parallel processing of both sensory and motor signals in the somatosensory barrel cortex.

Future experiments with higher cellular resolution are necessary to characterize the distinct structural and functional properties of neuronal subclasses in the different layers of the barrel-related cortices. In particular, it will be important to determine which neuron types in layers 2/3, 5, and 6 of S1 form synaptic connections with which target neuron types in layer S2 or M1 and *vice versa*.

Pyramidal neurons in layer 2/3 and 5 target the contralateral S1 cortex *via* dense callosal axon projections (Olavarria et al., 1984; Larsen et al., 2007; Petreanu et al., 2007; Aronoff et al., 2010). Callosal projections also preferentially target septal rather than barrel areas (Olavarria et al., 1984). A ChR2-assisted circuit mapping study showed that L2/3 pyramids target predominantly L2/3, L5A, and L5B pyramids in the contralateral S1 cortex; only few connection with L6 neurons were found (Petreanu et al., 2007). Functional interactions between S1 cortices in the two hemispheres have been demonstrated because a chronic suppression of the activity in one hemisphere down-regulates sensory responses in the contralateral S1 barrel cortex (Li et al., 2005). However, it remains to be determined whether these interactions occur *via* corticocortical connections or involve subcortical regions.

Furthermore, there are also connections from S1 barrel cortex to the ventral orbital and the ipsi- and contralateral perirhinal cortex, a cortical region that is a crucial link between the neo- and the allocortex (Deacon et al., 1983) and thus contribute to the processing of tactile information in the hippocampus (Pereira et al., 2007). However, these connections occur at a much weaker density than those targeting the whisker-related M1 and S2 cortices (Welker et al., 1988; Aronoff et al., 2010). Neurons in both the ventral orbital and the perirhinal cortex project also back to the S1 cortex (Aronoff et al., 2010).

In particular pyramidal cells in layers 5 and 6 have been demonstrated to send axonal projections back to the thalamus (see above and Bourassa et al., 1995; Zhang and Deschênes, 1997, 1998; Veinante et al., 2000b; Alloway et al., 2003; Killackey and Sherman, 2003; Groh et al., 2008; Liao et al., 2010; Theyel et al., 2010). Because these neurons receive input from either the VPM or POm nucleus of the thalamus (or both) they may be elements of thalamocorticothalamic feedback loops; but see (Guillery and Sherman, 2011). While some of them target the same thalamic nucleus from which they receive synaptic input (e.g., L6A pyramids) others interdigitate different thalamic nuclei, e.g., L5B pyramids that receive VPM input and project to the dorsal part of POm. This connection is also involved in the interaction of S1 and S2 cortex (Theyel et al., 2010): action potentials in corticothalamic L5B pyramidal cells result in the efficient recruitment of POm relay neurons (*via* their giant presynaptic terminals; Groh et al., 2008) which in turn activate L4 neurons in the higher-order S2 cortex (Carvell and Simons, 1987; Spreafico et al., 1987; Alloway et al., 2000; Theyel et al., 2010). This stimulation was eliminated following an inhibition of the thalamic nucleus (Theyel et al., 2010) suggesting a corticothalamocortical pathway via layer 5B from S1 cortex to higher-order sensory cortices such as the S2 cortex. This could run in parallel to the corticocortical signal flow but the exact functional characteristics of and difference between these two pathways are so far unkown.

L6B pyramids may also be involved in this process because they innervate POm (Killackey and Sherman, 2003) and are likely to receive synaptic input from VPM *via* L6A pyramids. Thus, the whisker-related S1 and S2 cortex are interconnected by at least two different routes: a direct feedforward route from thalamus to S1 and from there to S2 *via* long-range axon collaterals of S1 neurons and through a corticothalamocortical feedback involving the POm. The exact cellular identity of the neurons involved in these pathways remains to be determined.

Besides the thalamus, excitatory neurons in the barrel-related S1 cortex project also to other subcortical targets such as the striatum (and through this region the basal ganglia) (Donoghue and Kitai, 1981; Welker et al., 1988; Gerfen, 1989; Cowan and Wilson, 1994; Alloway et al., 1998, 1999, 2006; Wright et al., 1999; Hoover et al., 2003; Aronoff et al., 2010), the superior colliculus (tectum) (Wise and Jones, 1977; Welker et al., 1988; Hoffer et al., 2005; Cohen et al., 2008; Aronoff et al., 2010; Cohen and Castro-Alamancos, 2010) and the pons (Welker et al., 1988; Legg et al., 1989; Leergaard et al., 2000, 2004; Schwarz and Möck, 2001; Aronoff et al., 2010), all of which are involved in the integration of motor performance, sensation and general behavior. Most of the neurons targeting these regions reside in the infragranular layers of the ipsilateral S1 cortex and here mostly in layer 5B; however, a small population of corticostriatally projecting supragranular pyramidal cells appears to exist (Gerfen, 1989; Wright et al., 1999). In addition to these subcortical target regions, there are also direct projections of axons originating in S1 cortex back to the ipsilateral PrV and the contralateral SpV trigeminal nuclei (Welker et al., 1988; Jacquin et al., 1990; Aronoff et al., 2010) suggesting that neurons in these first relay stations of the whisker-to-cortex pathway are under a very direct feedback modulation of the S1 barrel cortex. How these different regions in the whisker system are interconnected with one another (e.g., the thalamic nuclei with the striatum), to which other brain regions involved in somatosensation project. How they integrate and coordinate sensory and motor signals is not a subject this but of other excellent reviews (see Alloway, 2008; Fox, 2008; Bosman et al., 2011).

## Conclusion

On the basis of the available studies it can be stated that the barrel cortex has a very prominent vertical organization with a pronounced and spatially confined thalamocortical input and signal transformation to supragranular and to a lesser degree also to infragranular layers. This vertical organization is clearly visible, more so than in other sensory cortices. The readily discernible barrel structure and the largely vertical axonal projection of several of several neuronal cell types in the barrel-related column serves as evidence for this fact. In addition, thalamic afferents from VPM and POm have a barrel or septal-related projection into the S1 barrel cortex. All these structural features support the concept of vertical modules in cortical signal processing.

A barrel-related column is not a separate unit. Synaptic mechanisms such as the coordinated modulation of L2/3 and L5 pyramidal cells by the long-range collaterals of L2/3 pyramidal cells may serve to enhance the output of the most active barrel column. In addition, many interactions between cortical output neurons and neurons in other cortical and subcortical target regions show somatotopic arrangements suggesting a specific interaction between cortical columns in different cortical areas, particular the M1 and S2 cortex. Therefore, it is likely that there is a link between the structure and function of the S1 barrel column and other cortical areas. Thus, a cortical column is not merely a structural unit but may be the prerequisite of vertical signal transfer. Some functional properties of the barrel cortex such as the angular tuning have even been assigned to substructures such as sub-barrel domains.

The barrel cortex and its barrel-related columns show many interactions with cortical and subcortical brain regions. First of all, the available data on the neuronal connectivity suggests that signal processing in the S1 barrel cortex is far from being a purely serial and hierarchical process. Rather, neuronal connections in the S1 barrel cortex represent a distributed network that includes many parallel steps at which subcortical (thalamic) input occurs and which has many feedback controls (most notably through monosynaptic thalamocorticothalamic connections). This is also true for the different cortical areas such as the barrel-related M1 and S2 cortices that are involved in—often reciprocal—synaptic signaling. Not only do they receive direct input *from* neurons in S1 but also from subcortical structures via corticothalamocortical feed-foward circuits. In return, neurons in M1 and S2 cortices influence the activity of S1 cortex both directly through corticocortical or indirectly through cortico-thalamo-cortical axonal projections. While this does not necessarily negate the importance of vertical signal transformation it suggests that neurons in a barrel column show a large degree of interaction with neurons in other cortical and subcortical areas. Such synaptic interactions are not necessarily organized in vertical modules. Future studies are required to define the connectivity and function of the pre- and postsynaptic neurons in these pathways. Such studies will be essential in identifying the different functional roles of cortical columns in the barrel cortex of rodents.

Furthermore, the elucidation of the excitatory cortical connectivity is largely dependent on the knowledge of the types of excitatory neurons. In recent years (see above) it has become increasingly more evident that there is a large degree of diversity in excitatory neurons of the neocortex (in addition to the well-known diversity of inhibitory interneurons, see e.g., Ascoli et al., 2008) and that these different types of excitatory neurons have very distinct properties and functional roles in the cortical microcircuitry. For example in their target region and neuron specificity. We are just beginning to unravel this diversity and much more work needs to be done to understand how it impacts on our view on cortical connectivity not only in the barrel cortex but also with other cortical regions.

Finally, the synaptic activity in the barrel cortex (and those in other cortical areas) is highly dynamic because it fluctuates slowly between depolarized “up” states and hyperpolarized “down” states (Steriade et al., 1993; Cowan and Wilson, 1994; Petersen et al., 2003; Brecht et al., 2004; Haider et al., 2006; Waters and Helmchen, 2006; for reviews see Destexhe et al., 2003; Buzsaki and Draguhn, 2004); a point that has not been discussed here. These fluctuations are under the control of neuromodulators such as acetylcholine and noradrenaline, (Eggermann and Feldmeyer, 2009; Constantinople and Bruno, 2011) which are released during different behavioral states such as sleep, arousal, and attention. Neuromodulators show a cell-specific effect on neuronal cell types and synaptic connections (e.g., Eggermann and Feldmeyer, 2009), for example with respect to the release probability and synaptic efficacy. Synaptic networks in the barrel cortex and other cortical areas are therefore not stable but highly dynamic and the synaptic weight in cortical microcircuits may change considerably. For future studies of cortical connectivity such connection-specific changes should be taken into account if one wants to understand the cellular correlates during different behavioral states.

### Conflict of interest statement

The author declares that the research was conducted in the absence of any commercial or financial relationships that could be construed as a potential conflict of interest.
